# The Edinburgh CT and genetic diagnostic criteria for lobar intracerebral haemorrhage associated with cerebral amyloid angiopathy: model development and diagnostic test accuracy study

**DOI:** 10.1016/S1474-4422(18)30006-1

**Published:** 2018-03

**Authors:** Mark A Rodrigues, Neshika Samarasekera, Christine Lerpiniere, Catherine Humphreys, Mark O McCarron, Philip M White, James A R Nicoll, Cathie L M Sudlow, Charlotte Cordonnier, Joanna M Wardlaw, Colin Smith, Rustam Al-Shahi Salman

**Affiliations:** a Centre for Clinical Brain Sciences, The University of Edinburgh, Edinburgh, UK; b UK Dementia Research Institute at The University of Edinburgh, The University of Edinburgh, Edinburgh, UK; c Row Fogo Centre for Research into Ageing and the Brain, The University of Edinburgh, Edinburgh, UK; d Usher Institute of Population Health Sciences and Informatics, The University of Edinburgh, Edinburgh, UK; e Department of Neurology, Altnagelvin Hospital, Londonderry, UK; f Institute of Neuroscience and Institute for Ageing, Newcastle University, Newcastle-upon-Tyne, UK; g Newcastle upon Tyne Hospitals National Health Services Foundation Trust, Newcastle-upon-Tyne, UK; h Clinical Neurosciences, Clinical and Experimental Sciences, University of Southampton, Southampton, UK; i Université Lille, Inserm U1171, Degenerative and Vascular Cognitive Disorders, CHU Lille, Department of Neurology, Lille, France

## Abstract

**Background:**

Identification of lobar spontaneous intracerebral haemorrhage associated with cerebral amyloid angiopathy (CAA) is important because it is associated with a higher risk of recurrent intracerebral haemorrhage than arteriolosclerosis-associated intracerebral haemorrhage. We aimed to develop a prediction model for the identification of CAA-associated lobar intracerebral haemorrhage using CT features and genotype.

**Methods:**

We identified adults with first-ever intracerebral haemorrhage diagnosed by CT, who died and underwent research autopsy as part of the Lothian IntraCerebral Haemorrhage, Pathology, Imaging and Neurological Outcome (LINCHPIN) study, a prospective, population-based, inception cohort. We determined *APOE* genotype and radiologists rated CT imaging appearances. Radiologists were not aware of clinical, genetic, and histopathological features. A neuropathologist rated brain tissue for small vessel diseases, including CAA, and was masked to clinical, radiographic, and genetic features. We used CT and *APOE* genotype data in a logistic regression model, which we internally validated using bootstrapping, to predict the risk of CAA-associated lobar intracerebral haemorrhage, derive diagnostic criteria, and estimate diagnostic accuracy.

**Findings:**

Among 110 adults (median age 83 years [IQR 76–87], 49 [45%] men) included in the LINCHPIN study between June 1, 2010 and Feb 10, 2016, intracerebral haemorrhage was lobar in 62 (56%) participants, deep in 41 (37%), and infratentorial in seven (6%). Of the 62 participants with lobar intracerebral haemorrhage, 36 (58%) were associated with moderate or severe CAA compared with 26 (42%) that were associated with absent or mild CAA, and were independently associated with subarachnoid haemorrhage (32 [89%] of 36 *vs* 11 [42%] of 26; p=0·014), intracerebral haemorrhage with finger-like projections (14 [39%] of 36 *vs* 0; p=0·043), and *APOE* ɛ4 possession (18 [50%] of 36 *vs* 2 [8%] of 26; p=0·0020). A prediction model for CAA-associated lobar intracerebral haemorrhage using these three variables had excellent discrimination (c statistic 0·92, 95% CI 0·86–0·98), confirmed by internal validation. For the rule-out criteria, neither subarachnoid haemorrhage nor *APOE* ɛ4 possession had 100% sensitivity (95% CI 88–100). For the rule-in criteria, subarachnoid haemorrhage and either *APOE* ɛ4 possession or finger-like projections had 96% specificity (95% CI 78–100).

**Interpretation:**

The CT and *APOE* genotype prediction model for CAA-associated lobar intracerebral haemorrhage shows excellent discrimination in this cohort, but requires external validation. The Edinburgh rule-in and rule-out diagnostic criteria might inform prognostic and therapeutic decisions that depend on identification of CAA-associated lobar intracerebral haemorrhage.

**Funding:**

UK Medical Research Council, The Stroke Association, and The Wellcome Trust.

## Introduction

About 85% of spontaneous intracerebral haemorrhages have no underlying macrovascular cause and are attributed to small vessel disease, mostly arteriolosclerosis with or without cerebral amyloid angiopathy (CAA).^1^, ^2^ CAA affects cortical and leptomeningeal vessels and is only associated with lobar intracerebral haemorrhage,^3^, ^4^ whereas arteriolosclerosis can cause intracerebral haemorrhage anywhere in the brain.

Identification of CAA-associated intracerebral haemorrhage is important because it is associated with a higher risk of recurrent intracerebral haemorrhage and post-stroke dementia than arteriolosclerosis-associated intracerebral haemorrhage,^5^, ^6^ and might increase the risk of intracerebral haemorrhage in patients taking antithrombotic drugs.^7^ Criteria to rule out CAA underlying intracerebral haemorrhage would allow clinicians to be more confident about the use of antithrombotic drugs;^2^, ^7^ ruling in CAA underlying intracerebral haemorrhage would provide important prognostic information.

The MRI-based modified Boston criteria have excellent sensitivity and good specificity for CAA.^8^ However, MRI can be unsuitable for very unwell patients in the acute setting or for those patients with contraindications, such as non-MRI compatible implanted devices or claustrophobia, and might be unavailable particularly in low-income and middle-income countries where 75% of worldwide deaths due to haemorrhagic stroke occur.^9^

**Research in context**

**Evidence before this study**

We did a systematic review of studies on imaging features of lobar or cerebellar intracerebral haemorrhage with pathologically proven cerebral amyloid angiopathy (CAA) published in MEDLINE (from 1946 to Nov 1, 2016) and Embase (from 1974 to Nov 1, 2016) using comprehensive electronic search strategies combining terms “stroke”, “cerebrovascular disorders”, (brain$ or cerebr$ or intracerebr$) adj5 (h?emorrhag$ or h?ematoma$), “amyloid beta-protein”, “cerebral amyloid angiopathy”, “vascular amyloidosis”, “congo red”, “pathology, clinical”, “pathology”, “histo?patholog$”, “post?mortem$” and “autops$” with no language restriction. We identified 22 case series describing imaging features of lobar or cerebellar intracerebral haemorrhage accompanying histopathologically proven CAA. Overall, the study quality was poor, with small sample sizes, unclear definitions of predictor or outcome variables, and infrequent masking of study assessors. The most frequently reported CT features of CAA-associated intracerebral haemorrhage in 21 case series were subarachnoid extension and an irregular intracerebral haemorrhage border. No diagnostic test accuracy studies have been done. Although the modified Boston MRI criteria are widely used for the identification of CAA-associated intracerebral haemorrhage, no CT-based diagnostic criteria exist for patients who cannot tolerate or do not have access to MRI.

**Added value of this study**

This diagnostic test accuracy study minimised biases, used masking, assessed inter-rater and intra-rater agreement, standardised index test and reference standard, and adhered to recommended approaches for analysis. We were able to develop a highly discriminatory and well calibrated prediction model using subarachnoid haemorrhage and finger-like projections from intracerebral haemorrhage on CT, and *APOE* ɛ4 possession, which was internally validated. We identified clinically useful probability cutoffs and two sets of diagnostic criteria that can rule in or rule out CAA-associated lobar intracerebral haemorrhage.

**Implications of all the available evidence**

Both the Boston MRI and the Edinburgh CT-based diagnostic criteria for CAA-associated lobar intracerebral haemorrhage are now available. The Edinburgh sensitive rule-out criteria and specific rule-in criteria based on CT and *APOE* genotype are potentially widely applicable for diagnostic, prognostic, and therapeutic decisions in everyday clinical practice if MRI is contraindicated, intolerable, or unavailable. Future research is required to externally validate these diagnostic criteria and evaluate their clinical use.

Other tests that can diagnose CAA include CT, which is usually the first test to diagnose intracerebral haemorrhage, and *APOE* genotype. Subarachnoid haemorrhage on CT occurs with 82% (95% CI 69–93) of cases of intracerebral haemorrhage accompanied by histopathologically proven CAA.^10^ The presence of an *APOE* ɛ4 allele is the strongest genetic association with histopathologically confirmed sporadic CAA (odds ratio [OR] 2·67, 95% CI 2·31–3·08), and the association is dose dependent and occurs regardless of dementia comorbidity.^11^ However, the diagnostic use of CT features and *APOE* genotype—alone or in combination—is unknown.

We aimed to develop a multivariable prediction model for identifying lobar intracerebral haemorrhage associated with CAA using CT and genetic features, internally validate the model, and assess the diagnostic accuracy of different cutoffs to rule in and rule out CAA-associated intracerebral haemorrhage.

## Methods

### Study design and participants

We did a community-based inception cohort study of spontaneous intracerebral haemorrhage in the Lothian health board region of Scotland (the Lothian IntraCerebral Haemorrhage, Pathology, Imaging and Neurological Outcome [LINCHPIN] study).^12^ We prospectively identified all incident cases of intracerebral haemorrhage with multiple overlapping sources of case ascertainment.^1^ We included consecutive adult patients (aged ≥16 years) with first-ever intracerebral haemorrhage confirmed by CT. We excluded patients with recurrent intracerebral haemorrhage; exclusively extra-axial intracranial haemorrhage; and intracerebral haemorrhage secondary to trauma, macrovascular causes, structural causes, or haemorrhagic transformation of an ischaemic stroke.^1^ We collected demographics, medical history, and drug use at diagnosis of intracerebral haemorrhage data by interviewing patients or their families or carers at the time of presentation and reviewing primary care and hospital records.^1^

The Scotland A Research Ethics Committee (10/MRE00/23) approved LINCHPIN. We obtained written informed consent from all participants or their immediate next of kin when participants did not have mental capacity.

### Index tests

Two neuroradiologists (MAR and PMW) independently evaluated reformatted head CT images with a standardised pro forma derived from previous large-scale stroke studies (appendix).^13^ They assessed extra-axial haemorrhage (in the subarachnoid, subdural, or intra-ventricular spaces), finger-like projections (elongated extensions arising from the haematoma, longer than they are wide, regardless of whether they extended to the cortex or not [appendix], variably referred to as lobulated or multinodular appearance in others studies^10^, ^14^), and other radiographic features (appendix). All raters did all assessments masked to clinical, genetic, and pathological information. We used the initial ratings done by MAR for primary analyses.

For *APOE* genotype analysis, we obtained DNA from peripheral blood samples or cerebellar tissue stored in the LINCHPIN brain bank with standard methods described in the appendix. Investigators were masked to clinical, CT, and pathological features during DNA extraction and genotyping (appendix). We classified *APOE* genotype as *APOE* ɛ2 possession if participants had at least one ɛ2 allele or *APOE* ɛ4 possession if they had at least one ɛ4 allele.

### Reference test

One neuropathologist (CS) assessed post mortem brain tissue according to a standard operating procedure (appendix). The maximum interval from death to autopsy was 5 days. The same neuropathologist rated CAA features (appendix) with a consensus rating scale,^15^ which rates the presence and severity of parenchymal and meningeal CAA (0–3); the presence of capillary CAA (0 or 1), and vasculopathy (0–2). Two neuropathologists (CS and CH) rated the presence and severity of non-CAA (or other small vessel disease) features in the left cerebral hemisphere using haematoxylin and eosin staining with a modified version of a published scale:^16^ none (very mild, occasional arteriolosclerosis without media splitting or luminal narrowing), mild (widespread mild or focal moderate arteriolosclerosis), moderate (widespread moderate or focal severe arteriolosclerosis, with splitting of the media and narrowing of the lumen), or severe (widespread severe arteriolosclerosis, fibrinoid necrosis, lipohyalinosis, evidence of vascular occlusion with or without recanalisation; appendix). We made the CAA ratings similar to the other small vessel disease ratings by restricting them to the parenchymal CAA scores in the left cerebral hemisphere, which we summed and graded for a CAA burden category (0=none, 1–4=mild, 5–8=moderate, and 9–12=severe). Macroscopic neuropathological assessment could not be masked to intracerebral haemorrhage location, but investigators were masked to other CT features, clinical information, and genotype. For microscopic histopathological assessment, investigators were masked to intracerebral haemorrhage location when possible (ie, unless the intracerebral haemorrhage was included in one of the prespecified sampled regions), as well as other CT features, clinical information, and genotype.

### Statistical analysis

We were unable to calculate the sample size required for a diagnostic test accuracy study because a systematic review^10^ identified only two retrospective cross-sectional studies that compared CT features of intracerebral haemorrhage associated with histopathologically proven CAA with intracerebral haemorrhage without CAA, but these two studies were at high risk of selection and information biases. Therefore, after completing recruitment to this prospective population-based study,^1^ we used the largest sample size possible (n=110) from its nested brain bank and we restricted models to three predictors (nine outcomes per variable) to reduce overfitting.^17^ We did a post-hoc power calculation using a two-sided *Z* test for a logistic regression model with a 5% level of significance, with subarachnoid haemorrhage as the main predictor (OR 10·9) and adjusting for the effect of other covariates (Nagelkerke *R*^2^ 0·06); this calculation showed that our maximum achievable sample size of 62 participants with lobar intracerebral haemorrhage would result in 70·6% power.

No data were missing from this study. We did statistical analyses using the *R* statistic package (version 3.3.2), with the exception of post-hoc power calculation and diagnostic accuracy statistics (sensitivity, specificity, likelihood ratios, and predictive values and their 95% CIs) which we obtained using G*Power 3.1.9.2 and VassarStats Clinical Calculator 1, respectively.

### Prediction modelling and performance

The multivariable prediction model aimed to use CT features and *APOE* genotype to predict lobar intracerebral haemorrhage that was associated with CAA, defined as a CAA burden category of moderate or severe, with or without other small vessel disease.

We assessed intra-rater and inter-rater agreement of CT features using unweighted Cohen's κ coefficient for categorical data, linear-weighted Cohen's κ coefficient for ordinal data, and intra-class correlation coefficient for continuous data. We excluded radiographic features with κ less than 0·5 from further analyses. We compared the distributions of clinical, genetic, and CT characteristics in cases of lobar intracerebral haemorrhage with or without moderate or severe CAA using χ^2^ test (or Fisher's exact test, where appropriate) for categorical variables and the Mann-Whitney *U* test for non-normally distributed continuous variables. For the full model we prespecified subarachnoid haemorrhage and *APOE* ɛ4 possession on the basis of systematic review data.^10^, ^11^ We included finger-like projections from the intracerebral haemorrhage on the basis of the strong univariate association that we found with CAA-associated lobar intracerebral haemorrhage. We used Firth's penalised likelihood logistic regression to assess the association of predictors with CAA-associated lobar intracerebral haemorrhage and calculate OR with 95% CIs, because finger-like projections showed complete separation between the two outcome groups.^18^ We assessed overall performance using Nagelkerke's *R*^2^, the Brier score, and the Akaike information criterion.^19^ We evaluated model discrimination with the concordance (c) statistic and discrimination slope, and displayed the discrimination graphically using a receiver operating characteristic plot. We assessed model calibration with the Hosmer–Lemeshow goodness-of-fit test and calibration plots. We used bootstrapping to evaluate the internal validity of the model performance measures.^20^ We used 2000 random bootstrap samples with replacement from the full sample of participants with lobar intracerebral haemorrhage, constructed models on these bootstrap samples, and derived optimism-adjusted performance measures to provide a realistic estimate of future performance.^19^

### Development of diagnostic criteria

We defined three risk categories for CAA-associated lobar intracerebral haemorrhage according to the probability of CAA-associated intracerebral haemorrhage predicted by the model: low (≤7%), medium (44–64%), and high (≥95%). We used decision curve analysis, which assesses the use of different risk category cutoffs across the full range of threshold probabilities and false positive and false negative weighting, to confirm the optimum risk category cutoffs for ruling CAA-associated intracerebral haemorrhage either in or out.^21^ We evaluated the sensitivity, specificity, positive and negative likelihood ratios, and predictive values and their 95% CIs for the diagnostic criteria that distinguished low versus medium or high risk categories, and high versus medium or low risk categories.

### Data sharing

Clinical, radiographic, genetic, and pathological data used in this study are available online, along with the code for logistic regression and internal validation.

### Role of the funding source

The funders of the study had no role in study design, data collection, data analysis, data interpretation, or writing of the report. The corresponding author had full access to all the data in the study and had final responsibility for the decision to submit for publication.

## Results

Between June 1, 2010, and Feb 10, 2016, 110 participants underwent research autopsy after non-contrast head CT that initially diagnosed first-ever intracerebral haemorrhage, after unavoidable exclusions (appendix).^12^ The median age was 83 years (IQR 76–87) and 49 (45%) were men (appendix). DNA for *APOE* genotyping was available for all 110 participants (peripheral blood sample for 28 participants and post-mortem cerebellar tissue for 82 participants). The median time from intracerebral haemorrhage to CT was 5 h (IQR 3–18) and the median time from CT to autopsy was 11 days (5–80). For most radiographic features, intra-rater agreement was substantial to almost perfect and inter-rater agreement was moderate to almost perfect (appendix).

**Figure 1:**
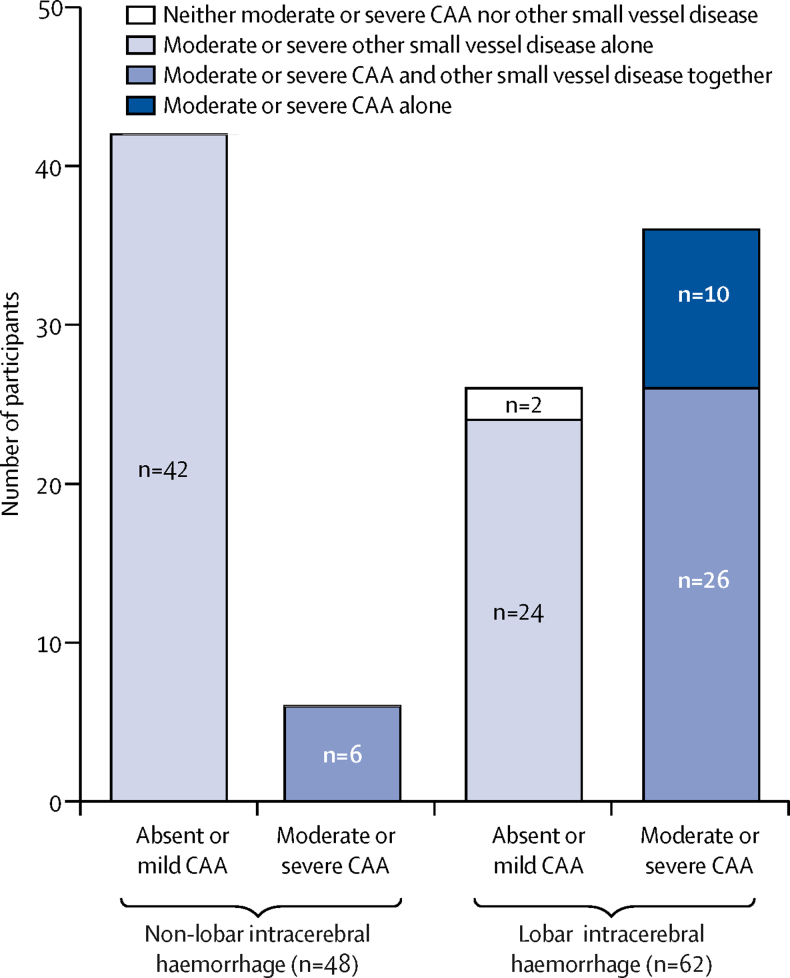
Pathological severity of CAA and other small vessel disease according to intracerebral haemorrhage location CAA=cerebral amyloid angiopathy.

62 (56%) participants had lobar intracerebral haemorrhage, 41 (37%) had deep intracerebral haemorrhage, and seven (6%) had infratentorial intracerebral haemorrhage (appendix). All 48 participants with non-lobar intracerebral haemorrhage had moderate or severe other small vessel disease: most (n=42 [88%]) had other small vessel disease with absent or mild CAA and six (13%) also had moderate or severe CAA, consistent with the population prevalence of CAA in octogenarians (figure 1, appendix).^2^ Therefore, we focused our further analyses on the prediction of moderate or severe CAA in participants with lobar intracerebral haemorrhage.

Of 62 participants with lobar intracerebral haemorrhage, 26 (42%) had moderate or severe other small vessel disease as well as moderate or severe CAA, 24 (39%) had moderate or severe other small vessel disease alone, ten (16%) had moderate or severe CAA alone, and two (3%) had no clear underlying cause of intracerebral haemorrhage (one participant had mild CAA and mild other small vessel disease, and the second participant had mild other small vessel disease and absent CAA, but neither had a macrovascular abnormality, coagulopathy, or tumour; figure 1). In univariable analyses, participants with lobar intracerebral haemorrhage and moderate or severe CAA were significantly more likely to be *APOE* ɛ4 carriers, and to have a strictly lobar intracerebral haemorrhage, subarachnoid haemorrhage, and finger-like projections from the intracerebral haemorrhage than participants with lobar intracerebral haemorrhage and absent or mild CAA (table 1).

**Table 1:** Characteristics of lobar intracerebral haemorrhage associated with severity of CAA

		**Absent or mild CAA (n=26)**	**Moderate or severe CAA (n=36)**	**Odds ratio (95% CI)**	**p value**
Age (years)		84 (78–88)	82 (79–85)	NC	0·41
Sex					
	Men	12 (46%)	11 (31%)	0·51 (0·18–1·46)	0·27
	Women	14 (54%)	25 (69%)	1·95 (0·68–5·55)	0·21
Hypertension		19 (73%)	23 (64%)	0·65 (0·22–1·96)	0·45
Antiplatelet use at intracerebral haemorrhage		15 (58%)	18 (50%)	0·73 (0·27–2·03)	0·55
Anticoagulant use at intracerebral haemorrhage		4 (15%)	5 (14%)	0·89 (0·21–3·68)	1·00
Dementia		2 (8%)	8 (22%)	3·43 (0·66–17·72)	0·17
*APOE* ɛ2 possession		3 (12%)	11 (31%)	3·37 (0·83–13·63)	0·077
*APOE* ɛ4 possession		2 (8%)	18 (50%)	12·00 (2·46–58·47)	0·0004
Multiple intracerebral haemorrhage		6 (23%)	3 (8%)	0·30 (0·07–1·35)	0·15
Left side		14 (54%)	18 (50%)	0·86 (0·31–2·35)	0·76
Intracerebral haemorrhage location					
	Frontal	10 (38%)	19 (53%)	1·79 (0·64–4·99)	0·27
	Parietal	6 (23%)	8 (22%)	0·95 (0·29–3·17)	0·94
	Temporal	5 (19%)	5 (14%)	0·68 (0·17–2·63)	0·57
	Occipital	5 (19%)	4 (11%)	0·53 (0·13–2·18)	0·38
Intracerebral haemorrhage volume (mL)		59 (23–126)	66 (22–117)	NC	0·72
Strictly lobar intracerebral haemorrhage		22 (85%)	36 (100%)	NA	0·027
Intraventricular extension		14 (54%)	17 (47%)	0·77 (0·28–2·11)	0·61
Any subarachnoid haemorrhage		11 (42%)	32 (89%)	10·91 (2·98–39·96)	<0·0001
Subdural extension		5 (19%)	7 (19%)	1·01 (0·28–3·64)	0·98
Midline shift		18 (69%)	21 (58%)	0·62 (0·21–1·80)	0·38
Finger-like projections		0	14 (39%)	34·16 (1·93–605·23)	0·0003
Cortical involvement		21 (81%)	35 (97%)	8·33 (0·91–76·28)	0·074
Dilute or seeping		9 (35%)	15 (42%)	1·35 (0·47–3·84)	0·59
Old vascular lesion		8 (31%)	15 (42%)	1·61 (0·55–4·66)	0·38
Anterior WML		··	··	··	0·26
	0	2 (8%)	8 (22%)	··	··
	1	16 (62%)	21 (58%)	··	··
	2	8 (31%)	7 (19%)	··	··
Posterior WML		··	··	··	0·65
	0	7 (27%)	6 (17%)	··	··
	1	3 (12%)	6 (17%)	··	··
	2	16 (62%)	24 (67%)	··	··
Central atrophy		··	··	··	0·26
	0	9 (35%)	10 (28%)	··	··
	1	17 (65%)	22 (61%)	··	··
	2	0	4 (11%)	··	··
Cortical atrophy		··	··	··	0·37
	0	4 (15%)	11 (31%)	··	··
	1	15 (58%)	18 (50%)	··	··
	2	7 (27%)	7 (19%)	··	··

Data are n (%) or median (IQR). CAA=cerebral amyloid angiopathy. NC=not calculable because the data are continuous. NA=not available as one or more cells contained a zero. WML=white matter lesion.

The multivariable prediction model for CAA-associated lobar intracerebral haemorrhage included three predictors: *APOE* ɛ4 possession, subarachnoid haemorrhage, and finger-like projections (table 2). The model calculates the predicted probability of moderate or severe CAA as follows (the predictor values are 1 when present and 0 when absent):

**Table 2:** Multivariable Firth's logistic regression prediction model for lobar intracerebral haemorrhage associated with moderate or severe cerebral amyloid angiopathy

	**β coefficient (SE)**	**Odds ratio (95% CI)**	**p value**
Intercept	−2·55 (0·89)	··	0·0040
*APOE* ɛ4 carrier	3·11 (1·01)	22 (4–862)	0·0020
Subarachnoid haemorrhage	2·31 (0·94)	10 (2–299)	0·014
Finger-like projections	3·20 (1·58)	27 (3–not reached)	0·043

$$\begin{array}{l} \text{Predicted}\;\text{probality}=\frac{1}{1+\exp^{-\text{risk}\;\text{score}}}\\ \text{Risk}\;\text{score}=-2\cdot 55+3\cdot 11\times (APOE\;ɛ4\;\text{positive})+2\cdot 31\times (\text{subarachnoid}\;\text{haemorrhage})+3\cdot 20\times (\text{finger-like}\;\text{projections}) \end{array}$$

All three predictors were independently associated with moderate or severe CAA. The variance inflation factor values (*APOE* ɛ4 carrier 1·33, subarachnoid haemorrhage 1·34, and finger-like projections 1·03) confirmed no evidence of multicollinearity between predictors. We did a sensitivity analysis excluding participants taking oral anticoagulants (n=9), given the high frequency of finger-like projections in such cases,^22^ which showed similar significance, direction, and magnitude of the independent associations (appendix).

**Figure 2:**
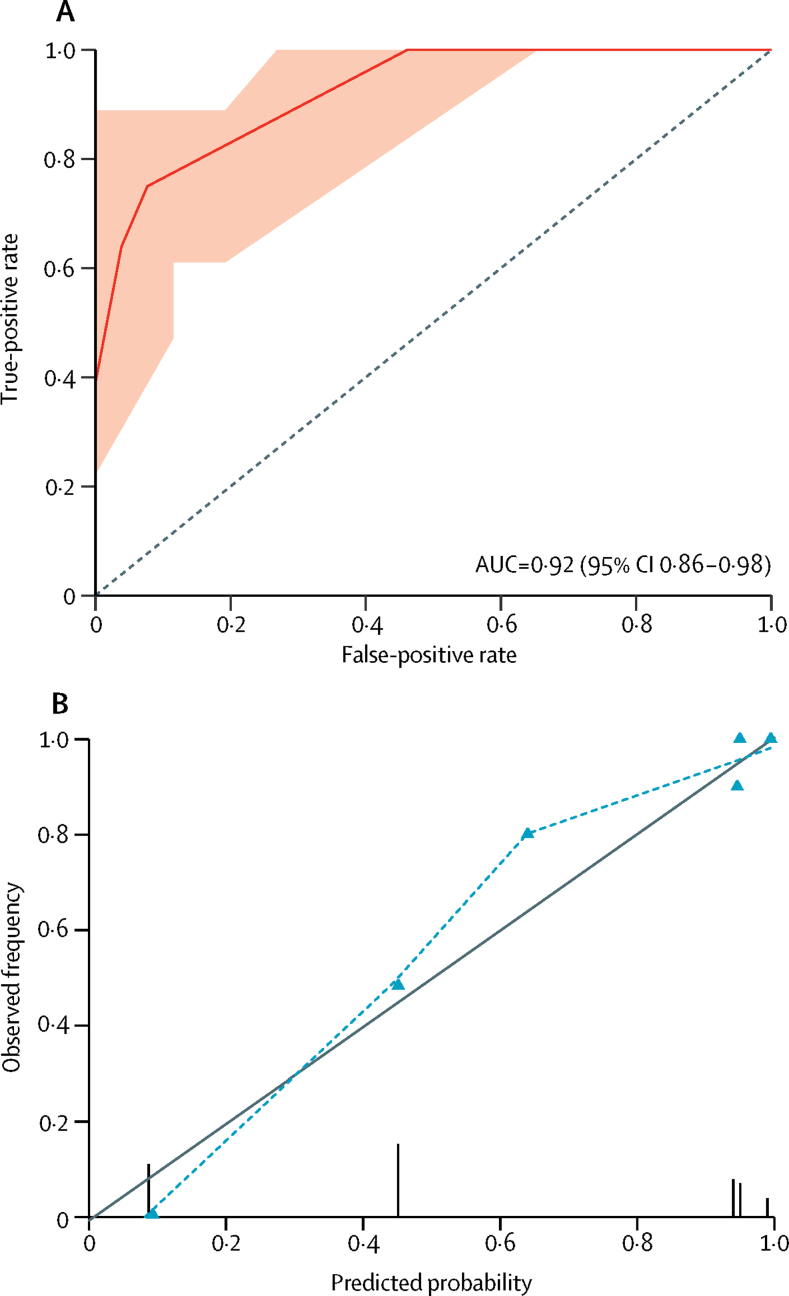
Discrimination and calibration measures of prediction model performance (A) Receiver operating characteristic curve for predicted probability of moderate or severe CAA. The AUC is equivalent to the c statistic. The shaded area represents the 95% CI of the AUC based on 2000 bootstrap replicates. The dotted line indicates a non-informative AUC of 0·50 for comparison. (B) Calibration plot of predicted probability versus observed frequency of moderate or severe CAA. Grey line indicates perfect calibration, the model's calibration is shown by the dotted line. Triangles represent the six different moderate or severe CAA risk groups produced by the prediction model. Vertical lines represent the frequency and distribution of model predicted probabilities. CAA=cerebral amyloid angiopathy. AUC=area under the curve.

The model showed excellent discrimination (c statistic 0·92, 95% CI 0·86–0·98) with no evidence of poor calibration (Hosmer–Lemeshow goodness of fit test p=0·685; figure 2, appendix). Internal validation identified small differences in the overall performance measures (eg, the Brier score increased from 0·11 to 0·12 and the c statistic decreased from 0·92 to 0·91), with no evidence of poor calibration (appendix).

We used the multivariable model to select two cutoff points to stratify the probability of moderate or severe CAA into low, medium, or high risk (appendix). When no predictors were present (low risk), the predicted probability of moderate or severe CAA was 7%. Subarachnoid haemorrhage or *APOE* ɛ4 possession in isolation (medium risk) predicted 44–64% probability of moderate or severe CAA. The presence of subarachnoid haemorrhage and at least one other predictor (high risk) predicted a probability of moderate or severe CAA of 95% or more.

**Figure 3:**
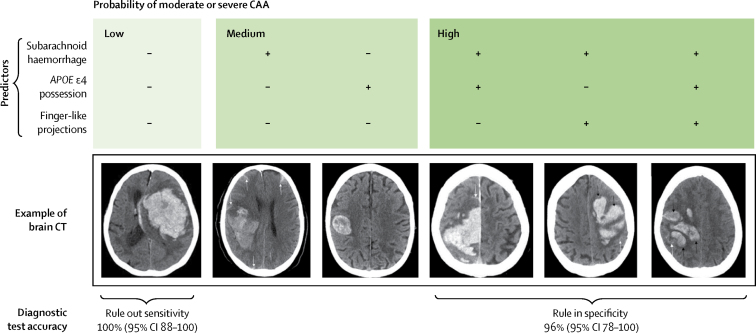
Categorisation of probability of lobar intracerebral haemorrhage associated with moderate or severe cerebral amyloid angiopathy according to the three predictor variables, with example CT images CAA=cerebral amyloid angiopathy. Adapted from Salman and Rodrigues (Creative Commons 4.0).^23^

Guided by how the predictors stratified the probability of moderate or severe CAA associated with lobar intracerebral haemorrhage, we identified two sets of diagnostic criteria including the three predictors (appendix, figure 3). Subarachnoid haemorrhage or *APOE* ɛ4 possession separated low from medium or high probability groups (appendix), and had a sensitivity of 100% (95% CI 88–100; appendix) meaning that the absence of these two predictors with lobar intracerebral haemorrhage ruled out moderate or severe CAA. Subarachnoid haemorrhage and *APOE* ɛ4 possession or finger-like projections, separated high from low or medium probability groups (appendix), and had a specificity of 96% (95% CI 78–100; appendix) meaning that the presence of these predictors with lobar intracerebral haemorrhage ruled in moderate or severe CAA. These optimum risk category cutoffs for ruling CAA-associated intracerebral haemorrhage either in or out were confirmed by decision curve analysis (appendix).

We repeated the prediction modelling and derived diagnostic criteria using CT ratings from a second independent investigator (PMW). The prediction model shows similar direction and magnitude of the predictors with moderate or severe CAA, while the sensitivity of the rule-out criteria remained 100% and specificity of the rule-in criteria increased to 100% (appendix).

We did a comparison of our CT and genetic criteria with the modified Boston criteria^8^ in the seven participants with lobar intracerebral haemorrhage who subsequently had MRI done within 6 months of the intracerebral haemorrhage, and found that all cases at high or intermediate probability of having moderate or severe CAA by our Edinburgh criteria were classified as probable CAA by the Boston criteria (appendix).

Since *APOE* genotyping might not be available worldwide, we assessed the diagnostic test accuracy of a simplified model on the basis of CT features alone (appendix). Subarachnoid haemorrhage alone had a sensitivity of 89% (95% CI 73–96) and the combination of subarachnoid haemorrhage and finger-like projections had 100% (95% CI 84–100) specificity (appendix). However, the inclusion of *APOE* ɛ4 improved the model (full model χ^2^=59·0, Akaike information criterion=49·9 *vs* simplified CT-based model χ^2^=41·9, Akaike information criterion=65·0, p<0·0001).

## Discussion

In our study, we used a systematically acquired brain tissue bank nested within a prospective, population-based cohort study to develop and internally validate a simple, three-variable model using two CT features (subarachnoid haemorrhage and finger-like projections from intracerebral haemorrhage) and *APOE* genotype to predict moderate or severe CAA associated with lobar intracerebral haemorrhage. The model had excellent discrimination and calibration. The diagnostic criteria might inform estimates of prognosis and decisions about antithrombotic drugs after intracerebral haemorrhage.

We identified two clinically useful diagnostic cutoffs that have implications for clinical practice. Neither subarachnoid haemorrhage nor *APOE* ɛ4 possession was 100% sensitive for moderate or severe CAA with a negative likelihood ratio of 0; a negative likelihood of less than 0·1 means a negative test is good at ruling out a diagnosis.^24^ Therefore, the absence of these features can rule out CAA-associated lobar intracerebral haemorrhage, which might identify people with a lower risk of recurrent intracerebral haemorrhage,^5^, ^25^ dementia,^6^ and susceptibility to the effects of antithrombotic drugs.^7^ The presence of subarachnoid haemorrhage and *APOE* ɛ4 possession or finger-like projections was 96% specific with a positive likelihood ratio 16·6. A positive likelihood of more than 10 means a positive test is good at ruling in a diagnosis,^24^ so the presence of these features can effectively rule in CAA-associated lobar intracerebral haemorrhage to identify patients for studies of CAA treatment.

Our diagnostic criteria are based on features identified on non-contrast CT, a widely available, relatively inexpensive diagnostic test with few contraindications, which is suitable in acutely unwell patients. The intra-rater and inter-rater agreement for subarachnoid haemorrhage and finger-like projections were moderate to almost perfect. Although *APOE* genotyping using peripheral blood samples is not a universally available test, the inclusion of *APOE* ɛ4 possession significantly improves the prediction model. Given the drive towards stratified medicine, these techniques are becoming increasingly cost-effective.^26^ Our criteria could be used in other patient groups to help guide the use of more restricted clinical resources by identifying patients who might benefit from advanced imaging, such as MRI or PET to assess for further features of CAA and other small vessel disease biomarkers.

This study minimised selection bias by using prospective case ascertainment in one community and inviting all potentially eligible people to consent to the nested brain bank. We minimised information bias by standardising imaging format; defining and systematically assessing radiographic features; standardising brain tissue acquisition; extensively sampling brain tissue, rather than using cortical biopsy; systematically assessing pathological features; using validated rating scales;^15^, ^16^, ^27^ and masking assessors. No data were missing and inter-rater agreement was moderate to substantial for the key predictors. We chose logistic regression rather than machine-learning approaches, such as neural networks, for our prediction model, because of its simplicity, familiarity, and transparency. We reduced overfitting by restricting the multivariable model to three predictors, prespecified two of these predictors, and avoided stepwise methods of predictor selection due to the probable instability of selection related to the sample size. We did internal validation with bootstrapping to further reduce optimism in study performance measures.

This study has some limitations. Sample size was modest and the post-hoc power calculation shows that the study has 71% power and therefore is at risk of a type II error. Our sample size left us unable to develop criteria to distinguish lobar intracerebral haemorrhage associated with CAA alone, other small vessel disease alone, and mixed CAA and other small vessel disease, which could be clinically important. However, this study has the largest sample we could achieve over 6 years in a population of about 850 000 people, with roughly 50% consenting soon after an acute brain injury,^12^ which is comparable with other brain banks.^28^

Although we tried to limit selection bias, participants who underwent autopsy were older, had larger intracerebral haemorrhage, more frequent intraventricular extension, and more severe posterior white matter lucencies, and generally died soon after their intracerebral haemorrhage compared with participants who did not (appendix); these differences were inevitable in standard clinical practice, and indicate to whom our results are generalisable. Whilst the frequency of model predictors (*APOE* ɛ4 possession, subarachnoid haemorrhage and finger-like projections) and the distribution of risk categories did not vary between those participants who underwent autopsy and those participants who did not undergo autopsy (appendix), the applicability of our criteria to other intracerebral haemorrhage groups—such as younger patients with smaller intracerebral haemorrhage—is unclear.

We only identified finger-like projections in cases of lobar intracerebral haemorrhage with moderate or severe CAA, making them very specific for CAA-associated haemorrhage. However, finger-like projections are difficult to define, subjective, and potentially prone to observer variability. We used both written and pictorial definitions to improve reliability and showed substantial intra-rater and moderate inter-rater agreement. Furthermore, we repeated the modelling using ratings from a second independent investigator, which resulted in rule-out criteria with 100% sensitivity and rule-in criteria with 100% specificity for CAA-associated lobar intracerebral haemorrhage. Previous studies^10^ have reported that irregular and lobulated intracerebral haemorrhage borders are more frequently associated with CAA-associated lobar intracerebral haemorrhage. However, these features were not defined in the studies, making them difficult to relate to our findings.

Finally, CT features of intracerebral haemorrhage, such as subarachnoid haemorrhage and finger-like projections, will evolve with time. The timecourse for this evolution in intracerebral haemorrhage is unknown and how it might affect the diagnostic accuracy of these criteria is unclear. We would expect a reduction in sensitivity with time, however our rule-out criteria remain 100% sensitive despite including participants who had a CT scan up to 7 days after symptom onset.

We are planning an external validation study to assess the performance of the prediction model and diagnostic criteria in other settings, countries, ethnic groups, and patient populations, such as those patients with smaller intracerebral haemorrhage, and determine the reproducibility of predictor assessment by other investigators (Greenberg SM, Massachusetts General Hospital, Boston, MA, USA, personal communication). Further large, representative samples with a systematic reference standard based on autopsy could attempt to distinguish lobar intracerebral haemorrhage associated with CAA alone, other small vessel disease alone, and mixed CAA and other small vessel disease. Comparison of these CT and genetic criteria and the widely used modified Boston criteria^8^ against a pathological reference standard would be noteworthy. Studies of the clinical and economic impact of these criteria on prognosis and therapeutic decisions might quantify, and hopefully confirm, their effect on the outcome of this devastating disease.
