# Supplementary material for: The Edinburgh CT and genetic diagnostic criteria for lobar intracerebral haemorrhage associated with cerebral amyloid angiopathy: model development and diagnostic test accuracy study
Source: Lancet Neurol. 2018 Mar;17(3):232–40. doi: 10.1016/S1474-4422(18)30006-1 (PMC5818029; doi:10.1016/S1474-4422(18)30006-1)
Supplement: Supplementary appendix [file mmc1.pdf]

THE LANCET
Neurology

Supplementary appendix

This appendix formed part of the original submission and has been peer reviewed. We post it as supplied by the authors.

Supplement to: Rodrigues MA, Samarasekera N, Lerpiniere C, et al. The Edinburgh CT and genetic diagnostic criteria for lobar intracerebral haemorrhage associated with cerebral amyloid angiopathy: model development and diagnostic test accuracy study. *Lancet Neurol* 2018; published online Jan 10. http://dx.doi.org/10.1016/S1474-4422(18)30006-1.

Index test methods

CT head – reformatting

One trainee neuroradiologist (MAR) reformatted the first diagnostic non-contrast head CT scan performed after ICH onset into standard axial (parallel to a line linking the floor of the sella turcica to the fastigium of the fourth ventricle), coronal (parallel to the posterior surface of the brain stem) and sagittal (parallel to the interhemispheric fissure) planes, with standard slice thickness (5mm), spacing (3mm), and windowing (centre 35, width 80).

CT rating proforma

LINCHPIN CT RATING FORM

P1

LINCHPIN ID: ____________________ SCAN DATE: ____________________

READ DATE: ____________________ RATER INITIALS: ____________________

IMAGE SERIES: *Significant series (unenhanced 5mm Axial, coronal & sagittal reformats)*

Grade overall image quality: Poor Adequate Good

1.	Is there any sign of acute ischaemic change?	Y	N	IF NO, GO TO Q.2
a.	On which side of the brain is the ischaemia?	R	L	BOTH

2.	Is there any acute parenchymal haemorrhage?	Y	N	IF NO, GO TO Q.8
a.	Is the main haemorrhage most likely Haemorrhagic Transformation of an Infarct or Primary Intracerebral Haemorrhage?	HTI	PICH	IF HTI, GO TO Q.8

3.	Are there multiple sites of acute parenchymal haemorrhage?	Y	N	
a.	Characterize each separate acute parenchymal haemorrhage starting with the largest:			

	1	2	3	4	5
Left/Right/ Central					
Anatomic Location code(s) (site(s) are which the ICH is thought to be centred)					
Deep (D), Lobar (L), Mixed lobar & deep (M), Infrantentorial (IT) Uncertain (U)					

Anatomic Location Codes for haemorrhages

**LOBAR**		**DEEP**	
F	Frontal lobe (not basal ganglia)	BG	Basal ganglia (not thalamus)
TE	Temporal lobe	TH	Thalamus
PA	Parietal lobe	IC	Internal capsule
O	Occipital lobe	EC	External capsule
**INFRATENTORIAL**		WM	Deep & periventricular WM and corpus callosum
CE	Cerebellum		
BS	Brainstem (Midbrain, pons, medulla)		

LINCHPIN CT Rating form v.3 07/04/16 – Derived with permission from IST3/LSR

b.	Is there any intraventricular haemorrhage?	Y	N	
c.	Is there any subarachnoid extension?	Y	N	IF YES, GO TO 1D. IF NO, GO TO 1E
d.	If there is subarachnoid extension, is this adjacent to the ICH? Record If distant or both	Y	N	
e.	Is there any subdural extension?	Y	N	
f.	Is there any midline shift or herniation?	Y	N	

4.	Is there a blood/fluid level within any parenchymal haemorrhage? (NOT including an intraventricular fluid level; start with the largest first)				
	1	2	3	4	5
Blood or fluid level (Y/N)					

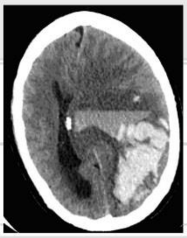

5.	Describe the border and shape of each separate acute parenchymal haemorrhage starting with the largest first (see examples below)				
	1	2	3	4	5
a. Irregular border? (Y/N)					
b. Finger-like protrusions? (Y/N)					
c. Round/oval? (Y/N)					
d. Reaches cortex? (Y/N)					

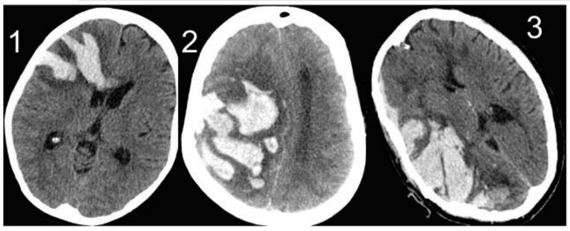

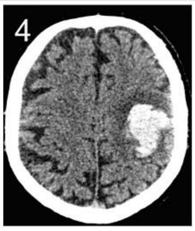

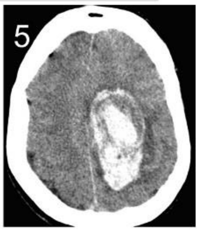

Images 1-3
Irregular border with **finger-like protrusions** to **cortex**

Images 4
Irregular border, reaches **cortex without** finger-like protrusions

Images 5
Regular border **without** finger-like protrusions & **does not** reach cortex

LINCHPIN CT Rating form v.3 07/04/16 – Derived with permission from IST3/LSR

6.	Describe the density of each acute parenchymal haemorrhage starting with the largest first

	1		2	3	4	5				
a. Variable density? (Y/N)	☐		☐	☐	☐	☐				
b. Dilute/seeping appearance? (Y/N)	☐		☐	☐	☐	☐				

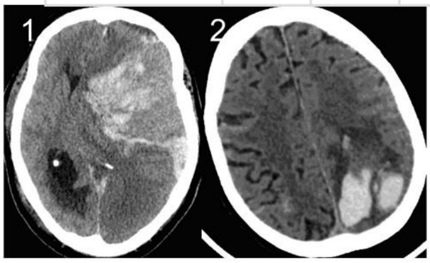

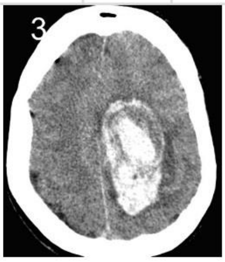

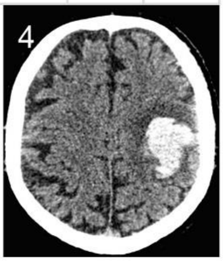

Variable density & dilute/seeping (1-2)
Blood in any single ICH has variable **density** & appears 'dilute'/seeping into adjacent brain tissue

Variable density (3)
Blood in any single ICH has variable **density** but not 'dilute'

Uniform density (4)
Blood in any single ICH has uniform **homogeneous density**

7.	Characterize the parenchymal haemorrhage(s) listed in 3 further. (Do not include extra-axial haemorrhage in the measurements)			
a.	What is the size of haemorrhage no.1 (mm)? A is largest diameter in the axial plane, B is longest axis perpendicular to A in the axial plane, C is maximum diameter in craniocaudal plane	A	B	C
b.	What is the size of haemorrhage no.2 (mm)?	A	B	C
c.	What is the size of haemorrhage no.3 (mm)?	A	B	C
d.	What is the size of haemorrhage no.4 (mm)?	A	B	C
e.	What is the size of haemorrhage no.5 (mm)?	A	B	C

8.	Are there any **old** vascular lesions?	Y	N	IF NO, GO TO Q.9
a.	Classify the old vascular lesions			

	A	B	C	D	E	F	G			
Code Y/N	☐	☐	☐	☐	☐	☐	☐			

A = old cortical infarct(s) B = old striatocapsular infarct(s) C = old borderzone infarct(s)

D = old lacunes E = old brainstem/cerebellar infarct(s) F = probable old **deep** haemorrhage

G = probable old **lobar** haemorrhage

9.	Classify any PERIVENTRICULAR LUCENCIES	[match "template" below]

diagram from van Swieten et al. JNNP 1990;53:1080-1083

Rating white matter lucency:
0= no lucency
1= lucency restricted to region adjoining ventricles
2= lucency covering entire region from lateral ventricle to cortex

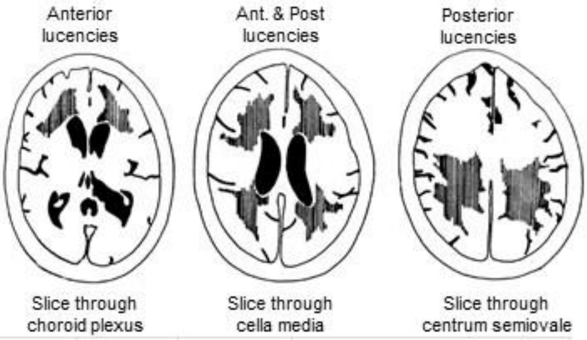

a.	Anterior white matter	0,1,2	Overall ☐
b.	Posterior white matter	0,1,2	Overall ☐

Exemplar:
AWM = 2 PWM = 1
Source:
http://www.sbirc.ed.ac.uk/documents/ctandmr%20reading%20form.pdf

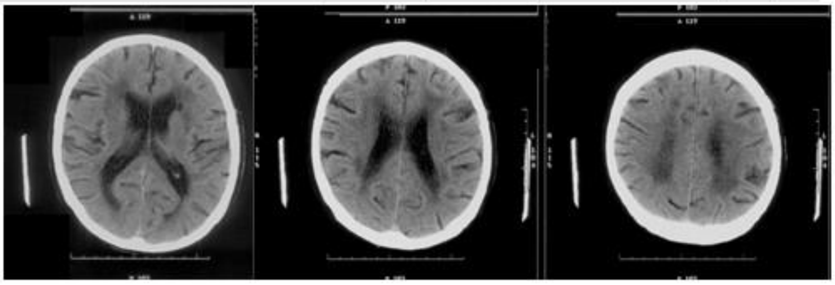

10	Rate any ATROPHY<br>0 = None. 1 = Moderate 2= Severe	[match "template" below]
a.	Central (deep)	Overall ☐

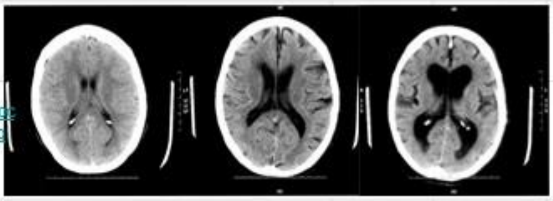

Source:
http://www.sbirc.ed.ac.uk/documents/ctandmr%20reading%20form.pdf

b.	Cortical	Overall ☐

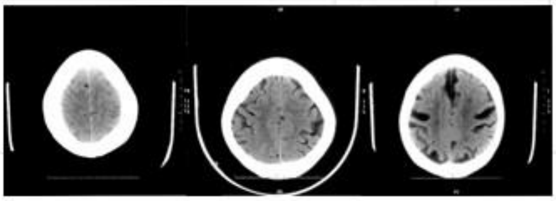

11	Comments

LINCHPIN CT Rating form v.3 07/04/16 – Derived with permission from IST3/LSR

12 separate case illustrations of finger-like projections

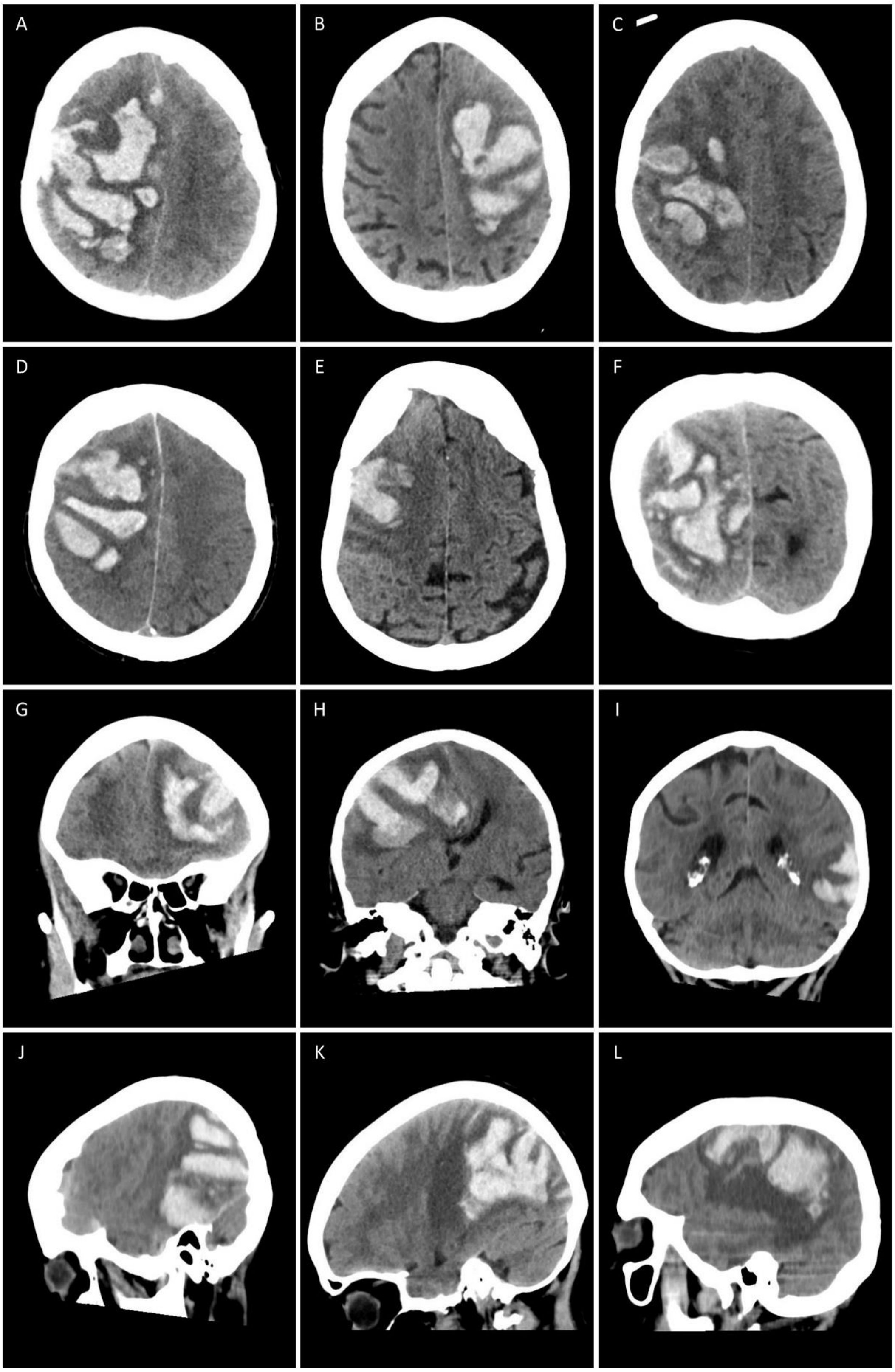

A-E: axial plane; F-I: coronal plane; J-L: sagittal plane

CT head – rating of other radiographic features

ICH volume was assessed using the *ABC/2* method where *A* is the largest diameter of ICH on in the axial plane (cm), *B* is the largest diameter at 90º to *A* on the same slice (cm), and *C* is the maximal cranio-caudal diameter (cm).[1]

ICH shape (e.g. irregular contour, finger-like projections) and appearance (e.g. fluid level and dilute/"seeping" density) was assessed.

We recorded the presence or absence of old infarcts, severity of anterior and posterior white matter lucencies using the van Swieten scale,[2] deep (enlargement of the ventricles) and superficial (enlargement of the sulci) cerebral atrophy using a template based three-point scale (absent/mild, moderate and severe).[3]

We used representative pictures to define specific imaging features – such as irregular contour, finger-like projection, dilute/"seeping" density and atrophy grades – which we defined as present or absent.

One trainee neuroradiologist (MAR) recorded ICH location using the Cerebral Haemorrhage Anatomical RaTing Scale (CHARTS),[4] and re-assessed the scans after a period of three months to evaluate intra-observer agreement.

DNA extraction and APOE genotyping

DNA was extracted from whole blood using a Nucleon Kit (GenProbe) with the BACC3 protocol. DNA samples were re-suspended in 1 ml TE buffer pH 7·5 (10mM Tris-Cl pH 7·5, 1mM EDTA pH 8·0). The yield of the DNA was measured using picogreen and normalised to 10ng/µl before genotyping. DNA was extracted from fresh-frozen brain tissue by homogenising using buffer ATL with proteinase K and incubating at 56ºC on a thermomixer at 1000 rpm then isolated using Qiagen DNeasy blood and tissue kit. DNA samples were re-suspended in 200µl of Qiagen elution buffer and normalised to 10ng/µl before genotyping. DNA was extracted from formalin fixed paraffin embedded tissue brain tissue using the Covaris E220 Focused Ultra Sonicator and the truXTRAC FFPE DNA kit, following the genomic DNA extraction protocol. 20µm tissue scrolls were deparaffinised by sonication for 2 x 5 minute periods before overnight incubation on a thermomixer at 56ºC with proteinase K. Crosslinking was reversed by incubation at 80ºC for 1 hour before purification in spin columns and elution in 50µL of Covaris Buffer BE (5mM Tris HCl pH 8·5).

Genotypes for two APOE single-nucleotide polymorphisms (rs429358 and rs7412) were determined using TaqMan single-nucleotide polymorphism genotyping assays (Applied Biosystems, Foster City, CA) on a ThermoFisher QuantStudio 12K Flex Real Time PCR System instrument with QuantStudio 12K Flex Software or Taqman Genotyper Software v1·3.

Reference test methods

Research post-mortem

The cerebral hemispheres were sectioned in the coronal plane at 1cm intervals, the first slice taken through the mammillary bodies. The cerebellum was sectioned in the sagittal plane and the brainstem axially. Tissue samples approximately 20x20x10mm were taken from each cerebral hemisphere from: frontal parasagittal cortex (BA9); Broca's area (BA44/45); temporal tip (BA38); caudate nucleus; basal ganglia; hippocampus; thalamus; frontal, temporal, parietal and occipital white matter; cerebellum; pons and medulla. Samples were bisected in the coronal plane, one block fixed in 10% unbuffered formalin for standard histological processing and the other frozen in nitrogen vapour.[5,6]

SVD assessment

We detected CAA in all cerebral and cerebellar lobes using immunohistochemistry with a monoclonal mouse antibody to human beta-amyloid, (Clone 6F/3D, Dako, Copenhagen) at a concentration of 1:100.

Example images of small vessel disease histopathological assessment grading

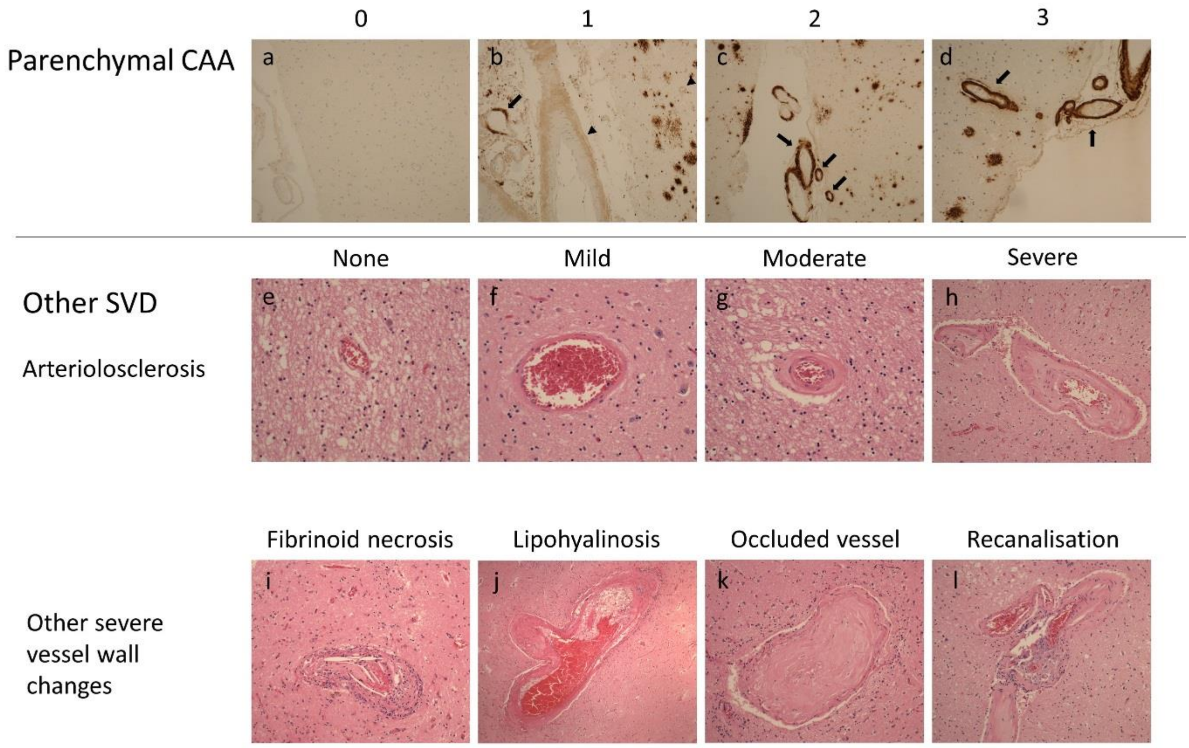

Flowchart of participants through the study

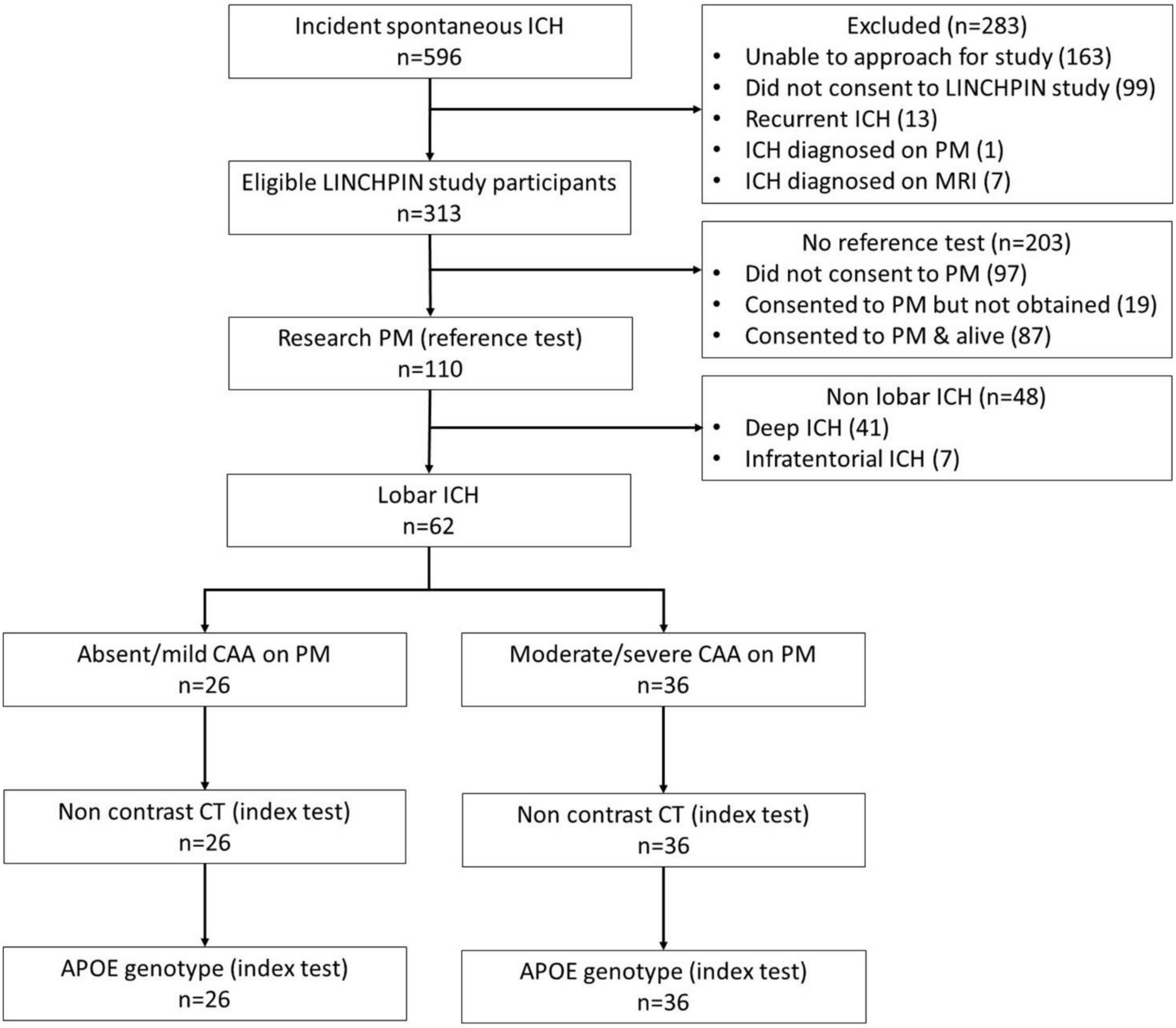

APOE = apolipoprotein. CAA = cerebral amyloid angiopathy. CT = computed tomography. ICH = intracerebral haemorrhage. LINCHPIN study = Lothian IntraCerebral Haemorrhage, Pathology, Imaging and Neurological Outcome study. MRI = magnetic resonance imaging. PM = research post-mortem.

Baseline characteristics of participants with lobar ICH who underwent post mortem versus those who did not

Characteristic	Post mortem (n=62)	No post mortem (n=86)	p value
**Median age, years**	83 (78-86)	78 (69-81)	0.00005
**Male**	23 (37)	36 (42)	0.56
**Hypertension**	42 (68)	45 (52)	0.06
**Antiplatelet use at ICH**	33 (53)	35 (41)	0.15
**Anticoagulant use at ICH**	9 (15)	14 (16)	0.75
**Dementia**	10 (16)	5 (6)	0.05
**APOE ε2+**	14 (23)	20 (33) ‡	0.19
**APOE ε4+**	20 (32)	25 (42)‡	0.28
**Multiple ICH**	9 (15)	5 (6)	0.23
**Left side**	32 (52)	47 (55)	0.71
**ICH location***			
• Frontal	29 (47)	36 (42)	0.91
• Parietal	14 (23)	23 (27)	
• Temporal	10 (16)	13 (15)	
• Occipital	9 (15)	14 (16)	
**Median ICH volume, cm^3**	60 (20-118)	20 (11-32)	0.000002
**Strictly lobar ICH**	58 (94)	85 (99)	0.16†
**IV extension**	31 (50)	17 (20)	0.0001
**Any SAH**	43 (69)	58 (67)	0.81
**Subdural extension**	12 (19)	14 (16)	0.63
**Midline shift**	39 (63)	36 (42)	0.01
**Finger-like projections**	14 (23)	23 (27)	0.56
**Cortical involvement**	56 (90)	74 (86)	0.43
**Dilute/seeping**	24 (39)	36 (42)	0.70
**Old vascular lesion**	23 (37)	29 (34)	0.67
**Anterior WML**			
0	10 (16)	22 (26)	
1	37 (60)	48 (56)	0.35
2	15 (24)	16 (19)	
**Posterior WML**			
0	13 (21)	34 (40)	
1	9 (14)	21 (24)	0.003
2	40 (65)	31 (36)	
**Central atrophy**			
0	19 (31)	34 (40)	
1	39 (63)	49 (57)	0.46†
2	4 (6)	3 (3)	
**Cortical atrophy**			
0	15 (24)	24 (28)	
1	33 (53)	46 (53)	0.79
2	14 (23)	16 (19)	
**CT CAA category**			
High	14 (23)	23 (27)	
Intermediate	29 (47)	35 (41)	0.74
Low	19 (31)	28 (33)	
**CT & APOE CAA category**			
High	24 (39)	23 (38)	
Intermediate	24 (39)	21 (35)	0.85
Low	14 (23)	16 (27)	

Data are number (%) or median (IQR). AF = atrial fibrillation. APOE ε2+ = apolipoprotein E ε2 allele present. APOE ε4+ = apolipoprotein E ε4 allele present. CAA = cerebral amyloid angiopathy. CT = computed tomography. ICH = intracerebral haemorrhage. SAH = subarachnoid haemorrhage. TIA = transient ischaemic attack. WML = white matter lucencies.* = presumed epicentre of haematoma defined by CHARTS. † = Fisher's exact test. ‡ 22 cases excluded as no APOE genotype

Intra- and inter-observer agreement for computed tomography features.

Characteristic	Frequency, n (%) (n=110)	Intra-observer agreement (95%CI) (n=110)	Inter-observer agreement (95%CI) (n=110)
**Multiple ICH**	12 (11)	1·00 (1·00-1·00)	0·64 (0·43-0·86)
**ICH side**		1·00 (1·00-1·00)	1·00 (1·00-1·00)
Left	55 (50)		
Right	51 (46)		
Central	4 (4)		
**Median ICH volume, cm^3 (IQR)**	31 (13-82)	0·98* (0·98-0·99)	0·96* (0·91-0·98)
**Supratentorial strictly lobar ICH**	58 (53)	0·98 (0·95-1·00)	0·42 (0·28-0·56)
**IV extension**	67 (61)	1·00 (1·00-1·00)	0·73 (0·60-0·86)
**Any SAH**	51 (46)	0·98 (0·95-1·00)	0·71 (0·58-0·84)
**Subdural extension**	12 (11)	0·96 (0·87-1·00)	0·59 (0·35-0·83)
**Midline shift**	66 (60)	0·75 (0·62-0·88)	0·61 (0·48-0·75)
**Blood/fluid level**	8 (7)	0·70 (0·41-0·98)	0·27 (-0·07-0·60)
**Irregular/lobulated**	69 (63)	0·68 (0·54-0·82)	0·13 (0·02-0·25)
**Finger-like projections**	16 (15)	0·72 (0·53-0·92)	0·60 (0·36-0·83)
**Round/oval**	36 (33)	0·72 (0·58-0·86)	0·05 (0·00-0·09)
**Cortical involvement**	60 (55)	0·98 (0·95-1·00)	0·80 (0·68-0·91)
**Dilute/seeping**	29 (26)	0·88 (0·78-0·98)	0·57 (0·39-0·75)
**Old vascular lesion**	48 (44)	0·95 (0·88-1·00)	0·56 (0·41-0·72)
**Anterior WML**		0·76† (0·65-0·88)	0·54† (0·40-0·67)
0	14 (13)		
1	66 (60)		
2	30 (27)		
**Posterior WML**		0·75† (0·66-0·84)	0·52† (0·40-0·64)
0	27 (25)		
1	20 (18)		
2	63 (57)		
**Central atrophy**		0·70† (0·58-0·82)	0·51† (0·37-0·64)
0	24 (22)		
1	66 (60)		
2	20 (18)		
**Cortical atrophy**		0·65† (0·53-0·78)	0·55† (0·41-0·69)
0	19 (17)		
1	64 (58)		
2	27 (25)		

Agreement assessed with un-weighted Cohen's kappa, linear-weighted kappa†, or intraclass correlation coefficient*. ICH = intracerebral haemorrhage. Cohen suggested the Kappa result be interpreted as follows: values ≤ 0 as indicating no agreement and 0.01–0.20 as none to slight, 0.21–0.40 as fair, 0.41– 0.60 as moderate, 0.61–0.80 as substantial, and 0.81–1.00 as almost perfect agreement. IV = intraventricular. SAH = subarachnoid haemorrhage. WML = white matter lesion.

Baseline characteristics of all ICH, stratified by location

	All ICH (n=110)		Lobar ICH (n=62)		Non-lobar ICH (n=48)	
**Median age at ICH, years**	83	(76-87)	83	(78-86)	82	(75-87)
**Male**	49	(45)	23	(37)	26	(54)
**Median time from ICH to CT, hours**	5	(3-18)	6	(3-29)	4	(3-15)
**Median time from CT to post-mortem, days**	11	(5-80)	11	(6-134)	9	(5-23)
**Previous ischaemic stroke/TIA**	23	(21)	12	(19)	11	(23)
**Coronary artery disease**	25	(23)	16	(26)	9	(19)
**Atrial fibrillation**	31	(28)	19	(31)	12	(25)
**Diabetes**	14	(13)	6	(10)	8	(17)
**Hypertension**	77	(70)	42	(68)	35	(73)
**Hyperlipidaemia**	15	(14)	8	(13)	7	(15)
**Antiplatelet use at ICH**	53	(48)	33	(53)	20	(42)
**Anticoagulant use at ICH**	20	(18)	9	(15)	11	(23)
**Dementia before ICH**	18	(16)	10	(16)	8	(17)
**APOE ε2+**	21	(19)	14	(23)	7	(15)
**APOE ε4+**	34	(31)	20	(32)	14	(29)
**ICH location***						
• Lobar	62	(56)	62	(100)		
○ Frontal	29	(26)	29	(47)		
○ Parietal	14	(13)	14	(23)		
○ Temporal	10	(9)	10	(16)		
○ Occipital	9	(8)	9	(15)		
• Deep	41	(37)			41	(85)
○ Basal ganglia	22	(20)			22	(46)
○ Thalamic	19	(17)			19	(40)
• Infratentorial	7	(6)			7	(15)
○ Brainstem	4	(4)			4	(8)
○ Cerebellum	3	(3)			3	(6)

Data are number (%) or median (IQR). AF = atrial fibrillation. APOE ε2+= apolipoprotein E ε2. allele present. APOE ε4+ = apolipoprotein E ε4 allele present. CT = computed tomography. ICH = intracerebral haemorrhage. TIA = transient ischaemic attack. * = presumed epicentre of haematoma defined by CHARTS[4]

Distribution of cerebral small vessel disease sub-types by ICH location

	Non-lobar ICH (n=48)		Lobar ICH (n=62)	
**Pathology Category**	*CAA absent*	*CAA present*	*CAA absent*	*CAA present*
Other SVD absent	0 (0)	0 (0)	0 (0)	0 (0)
Other SVD present	32 (67)	16 (33)	16 (26)	46 (74)
**Pathology Category**	*CAA absent/mild*	*CAA moderate/severe*	*CAA absent/mild*	*CAA moderate/severe*
Other SVD absent/mild	0 (0)	0 (0)	2 (3)	10 (16)
Other SVD moderate/severe	42 (88)	6 (13)	24 (39)	26 (42)

Data are number (%). CAA = cerebral amyloid angiopathy. ICH = intracerebral haemorrhage. SVD = small vessel disease

Logistic regression model with Firth correction fitted in participants with lobar ICH associated with moderate/severe CAA, excluding 9 cases taking oral anticoagulants at the time of ICH

	β Coefficient (standard error)	Odds ratio (95%CI)	p value
**Intercept**	-2·43 (0·92)		0·009
**APOE ε4 +**	2·77 (1·00)	15·97 (2·93-587·82)	0·006
**Subarachnoid haemorrhage**	2·18 (0·97)	8·89 (1·69-277·39)	0·02
**Finger-like projections**	3·09 (1·60)	21·97 (2·19-∞)	0·05

APOE ε4+ = apolipoprotein E ε4 allele present.

Performance measures of the diagnostic prediction model in the development dataset (n=62) and following internal validation using the same dataset (n=62; 2,000 bootstrap samples)

	Development	Internal validation
**Overall**		
Brier score	0·11	0·12
R^2 (Nagelkerke)	0·57	0·51
Akaike information criterion	49·90	55·90
**Discrimination**		
c statistic	0·92	0·91
Discrimination slope	0·52	0·50
**Calibration**		
Hosmer-Lemeshow test	χ^2 = 0·55, p = 0·76	χ^2 = 2·79, p = 0·25

Predicted and observed frequencies, and categorisation of the probability of lobar ICH associated with moderate/severe CAA according to the three predictor variables

Predictors present			Predicted risk of moderate/severe CAA, %	Observed frequency of moderate/severe CAA			Moderate/severe CAA probability
Subarachnoid haemorrhage	APOE ε4+	Finger-like projections			(%)	[95%CI]	
-	-	-	7	0/14	(0)	[0-22%]	**Low**
+	-	-	44	9/19	(47)	[27-68%]	**Medium**
-	+	-	64	4/5	(80)	[38-99%]	
+	+	-	95	9/10	(90)	[60-99%]	**High**
+	-	+	95	9/9	(100)	[70-100%]	
+	+	+	100	5/5	(100)	[57-100%]	

Cross tabulations of the Edinburgh CT and genetic diagnostic criteria for lobar ICH associated with moderate/severe CAA against the reference standard

Diagnostic criteria (index test)	Reference standard		
**Subarachnoid haemorrhage or APOE ε4+**	**Moderate/severe CAA at post-mortem**		
	Present	Absent	Total
Positive	36	12	48
Negative	0	14	14
Total	36	26	62

Diagnostic criteria (index test)	Reference standard		
**Subarachnoid haemorrhage and (APOE ε4+ or finger-like projections)**	**Moderate/severe CAA at post-mortem**		
	Present	Absent	Total
Positive	23	1	24
Negative	13	25	38
Total	36	26	62

APOE = apolipoprotein. CAA = cerebral amyloid angiopathy. CT = computed tomography.

Diagnostic test accuracy statistics for the two sets of Edinburgh CT and genetic diagnostic criteria for lobar ICH associated with moderate/severe CAA

	Edinburgh CT and genetic diagnostic criteria			
	Subarachnoid haemorrhage or APOE ε4+		Subarachnoid haemorrhage and (APOE ε4+ or finger-like projections)	
**Sensitivity**	100	(88-100)	64	(46-79)
**Specificity**	54	(34-73)	96	(78-100)
**Positive likelihood ratio**	2·2	(1·4-3·3)	16·6	(2·4-115·3)
**Negative likelihood ratio**	0	(0-NaN)	0·4	(0·2-0·6)
**Positive predictive value**	75	(60-86)	96	(77-100)
**Negative predictive value**	100	(73-100)	66	(49-80)
**Youden's Index**	0·54		0·60	

Data are percentage or ratio (95% confidence interval). APOE = apolipoprotein. CAA = cerebral amyloid angiopathy. CT = computed tomography. NaN = not a number – calculation cannot be performed because one of the values includes a zero

Decision curves of predictions and classifications of moderate/severe CAA in participants with lobar ICH using fixed cut-off points from the CT and APOE genotype prediction model to assign patients as positive or negative for moderate/severe CAA.

The threshold probability is the level of diagnostic certainty above which a patient or clinician would choose to be treated. Equally, it could be used by a researcher to identify patients eligible for inclusion in a randomised controlled trial or identify cases and controls for a case-control study. The threshold probability is low in situations where we want to avoid false negatives (e.g. when trying to rule out CAA-associated lobar ICH) and high when false positives are to be avoid (e.g. when trying to rule in CAA-associated lobar ICH). Net benefit is the difference between those expected to benefit (true positives identified using the strategy – expected benefit) and those expected to be harmed (false positives identified using the strategy multiplied by a weighting factor based on the threshold probability – expected harm). The curves which maximise net benefit represent the optimal strategy for the associated threshold probabilities. The solid black line indicates a policy of treating no one, the grey line a policy of treating all.

The combination of subarachnoid haemorrhage or APOEε4 is the best diagnostic strategy for low threshold probabilities (0-0.55), where harm of unnecessary treatment is limited and false negatives avoided (i.e. useful for ruling CAA-associated lobar ICH out). For high threshold probabilities (0.55-0.90), where there is harm of overtreatment and false positives should be avoided, the criteria of subarachnoid haemorrhage AND (APOE ε4 OR Finger-like projections) maximises net benefit (i.e. for ruling CAA-associated lobar ICH in).

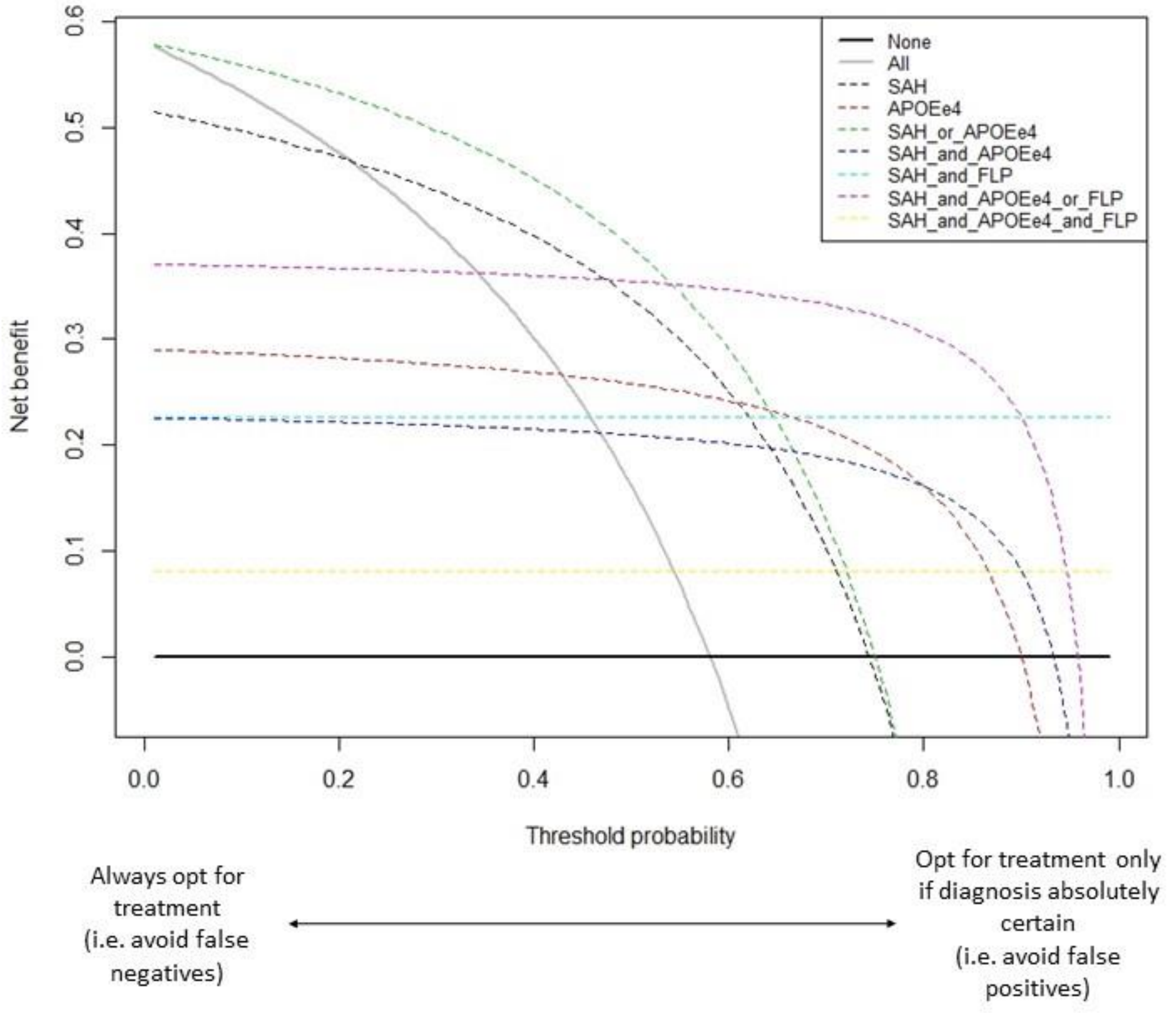

APOE ε4 = apolipoprotein E ε4 allele present. FLP = finger-like projection. SAH = subarachnoid haemorrhage

Logistic regression model using PMW ratings for CT-based features and APOE genotype, with Firth correction fitted in participants with lobar ICH associated with moderate/severe CAA.

	β Coefficient (standard error)		Odds ratio (95%CI)		p value
**Intercept**	-3·39	(1.46)			0·02
**APOE ε4 +**	4·18	(1.49)	65	(7-∞)	0·005
**Subarachnoid haemorrhage**	3·09	(1.47)	22	(3-∞)	0·04
**Finger-like projections**	3·01	(1.61)	20	(2-∞)	0·06

APOE ε4+ = apolipoprotein E ε4 allele present.

This model calculates the predicted probability of moderate/severe CAA as follows:

Predicted probability = $1/(1 + \exp^{-\text{ risk score}})$

Risk score = -3.39 + 4.18 x (APOEe4+) + 3.09 x (subarachnoid haemorrhage) + 3.01 x (finger-like projections)

The predictor values are one when present and zero when absent.

Discrimination and calibration measures of CT and APOE diagnostic prediction model performance using PMW CT ratings

A. Receiver operating characteristic (ROC) curve for predicted probability of moderate/severe CAA. The area under the curve (AUC) is equivalent to the c statistic. The shaded area represents the 95% confidence interval of the AUC based on 2,000 bootstrap replicates. The dotted line indicates a non-informative AUC of 0·50 for comparison. B. Calibration plot of predicted probability versus observed frequency of moderate/severe CAA. The grey line indicates perfect calibration, the model's calibration is shown by the dotted line. The triangles represent the six different moderate or severe CAA risk groups produced by the prediction model. The vertical lines along the x axis represent the frequency and distribution of model predicted probabilities.

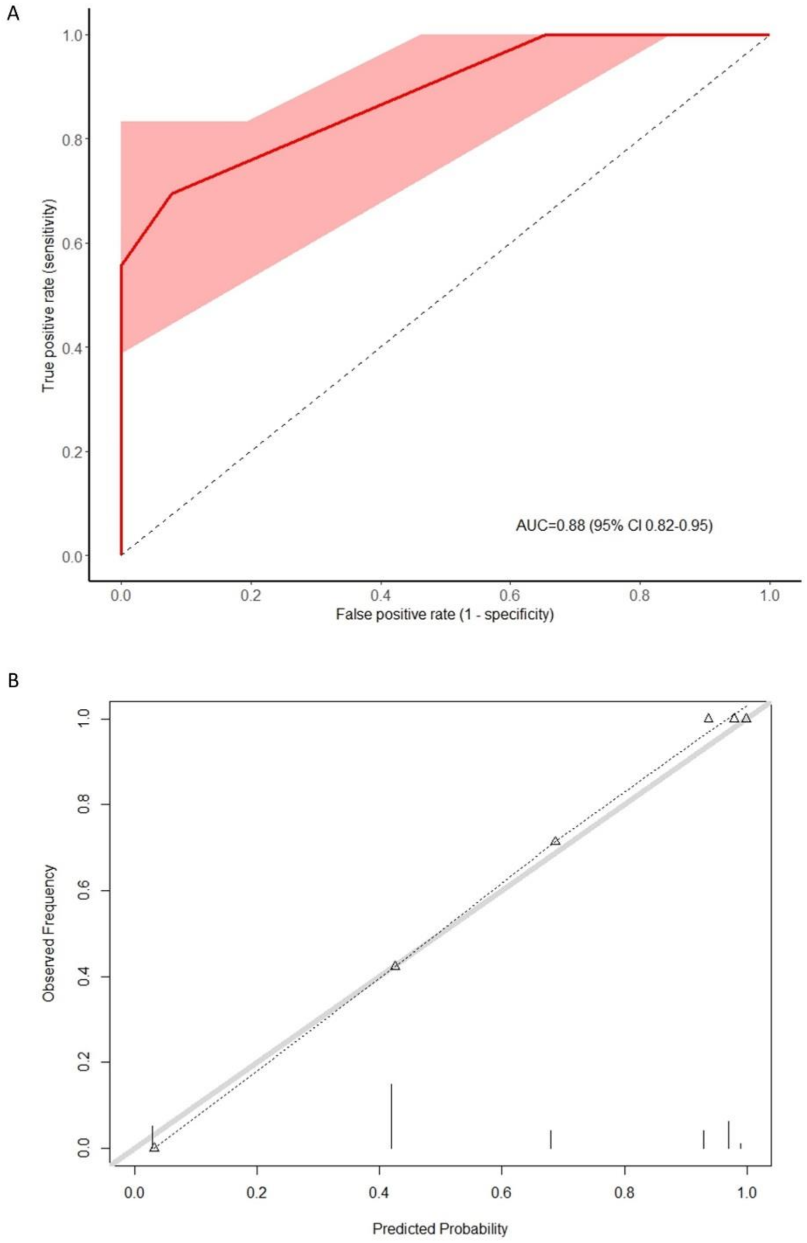

Performance measures of CT and APOE diagnostic prediction model using PMW CT ratings in the development dataset (n = 62) and following internal validation using the same dataset (n = 62; 2,000 bootstrap samples).

	Development	Internal validation
**Overall**		
Brier score	0.13	0.14
R^2 (Nagelkerke)	0.51	0.47
Akaike information criterion	53.78	57.86
**Discrimination**		
c statistic	0.88	0.88
Discrimination slope	0.45	0.43
**Calibration**		
Hosmer-Lemeshow test	χ^2 = 0·74, p = 0·86	χ^2 = 2.01, p = 0·37

Risk categories for lobar ICH associated with moderate/severe CAA using CT and APOE diagnostic criteria using PMW CT ratings

Predictors present			Predicted risk of moderate/severe CAA, %	Observed frequency of moderate/severe CAA (%) [95%CI]			Moderate/ severe CAA probability
Subarachnoid haemorrhage	APOE ε4+	Finger-like projections					
-	-	-	3	0/9	(0)	[0-30%]	Low
+	-	-	43	11/26	(42)	[26-61%]	Medium
-	+	-	69	5/7	(71)	[36-92%]	
+	+	-	98	11/11	(100)	[74-100%]	High
+	-	+	94	7/7	(100)	[65-100%]	
+	+	+	100	2/2	(100)	[34-100%]	

APOE ε4+ = apolipoprotein E ε4 allele present. CAA = cerebral amyloid angiopathy

Cross tabulations of the Edinburgh CT and genetic diagnostic criteria for lobar ICH associated with moderate/severe CAA using PMW CT ratings against the reference standard

**Diagnostic criteria (index test)**	**Reference standard**		
**Subarachnoid haemorrhage or APOE ε4+**	**Moderate/severe CAA at post-mortem**		
	Present	Absent	Total
Positive	36	17	53
Negative	0	9	9
Total	36	26	62
**Diagnostic criteria (index test)**	**Reference standard**		
**Subarachnoid haemorrhage and (APOE ε4+ or finger-like projections)**	**Moderate/severe CAA at post-mortem**		
	Present	Absent	Total
Positive	20	0	20
Negative	16	26	42
Total	36	26	62

APOE ε4+ = apolipoprotein E ε4 allele present. CAA = cerebral amyloid angiopathy

Diagnostic test accuracy statistics for the Edinburgh CT and genetic diagnostic criteria for lobar ICH associated with moderate/severe CAA using PMW CT ratings

	Edinburgh CT and genetic diagnostic criteria			
	Subarachnoid haemorrhage or APOE ε4+		Subarachnoid haemorrhage and (APOE ε4+ or finger-like projections)	
**Sensitivity**	100	(88-100)	56	(38-72)
**Specificity**	35	(18-56)	100	(84-100)
**Positive likelihood ratio**	1.5	(1.2-2.0)	∞	(NaN-∞)
**Negative likelihood ratio**	0	(0-NaN)	0.4	(0.3-0.6)
**Positive predictive value**	68	(54-80)	100	(80-100)
**Negative predictive value**	100	(63-100)	62	(46-76)
**Youden's Index**	0.35		0.56	

Data are percentage or ratio (95% confidence interval). NaN = not a number – calculation cannot be performed because one of the values includes a zero.

Comparison of the Edinburgh CT and genetic criteria with the modified Boston criteria in participants with lobar ICH who underwent MRI during life

Age at ICH	Modified Boston MRI classification	Edinburgh CT and genetic classification	Pathological CAA grade
79	Probable	High	Severe
76	Probable	High	Moderate
72	Probable	High	Moderate
84	Probable	Intermediate	Severe
90	Probable	Intermediate	Mild
83	Probable	Intermediate	Absent
83	Probable	Low	Absent

Logistic regression model using only CT-based features without APOE genotype, with Firth correction fitted in participants with lobar ICH associated with moderate/severe CAA.

	β Coefficient (standard error)	Odds ratio (95%CI)	p value
**Intercept**	-1·24 (0·55)		0·02
**Subarachnoid haemorrhage**	1·71 (0·67)	5·54 (1·63-26·09)	0·01
**Finger-like projections**	2·89 (1·54)	18·03 (2·04-∞)	0·06

This model calculates the predicted probability of moderate/severe CAA as follows:

Predicted probability = 1/(1 + $\exp^{-\text{ risk score}}$)

Risk score = -1·24 + 1·71 x (subarachnoid haemorrhage) + 2·89 x (finger-like projections)

The predictor values are one when present and zero when absent.

Discrimination and calibration measures of CT-based diagnostic prediction model without APOE genotype

A. Receiver operating characteristic (ROC) curve for predicted probability of moderate/severe CAA. The area under the curve (AUC) is equivalent to the c statistic. The shaded area represents the 95% confidence interval of the AUC based on 2,000 bootstrap replicates. The dotted line indicates a non-informative AUC of 0·50 for comparison. B. Calibration plot of predicted probability versus observed frequency of moderate/severe CAA. The grey line indicates perfect calibration, the model's calibration is shown by the dotted line. The triangles represent the three different moderate or severe CAA risk groups produced by the prediction model. The vertical lines along the x axis represent the frequency and distribution of model predicted probabilities.

A

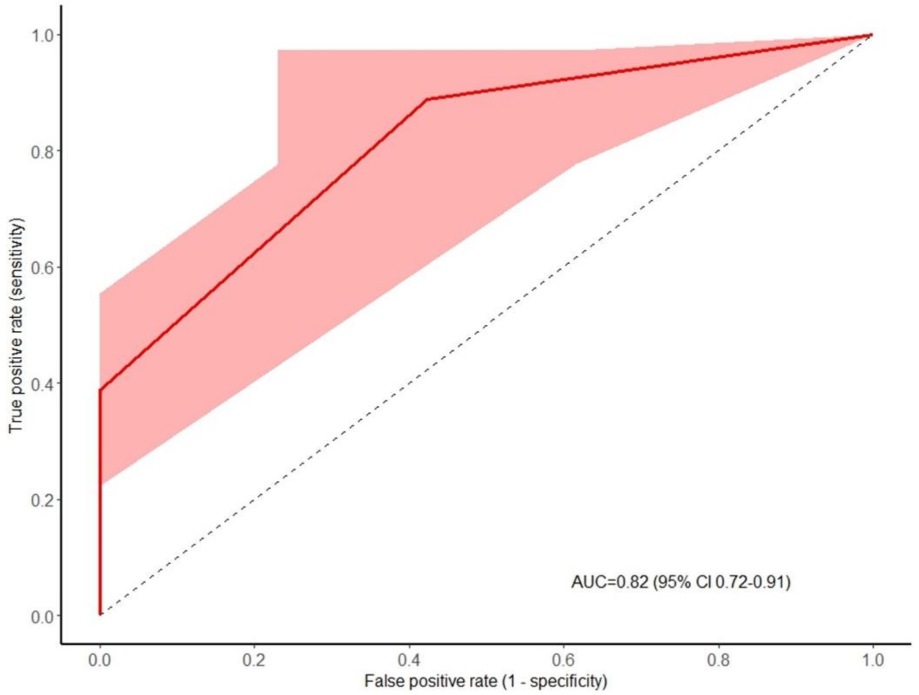

B

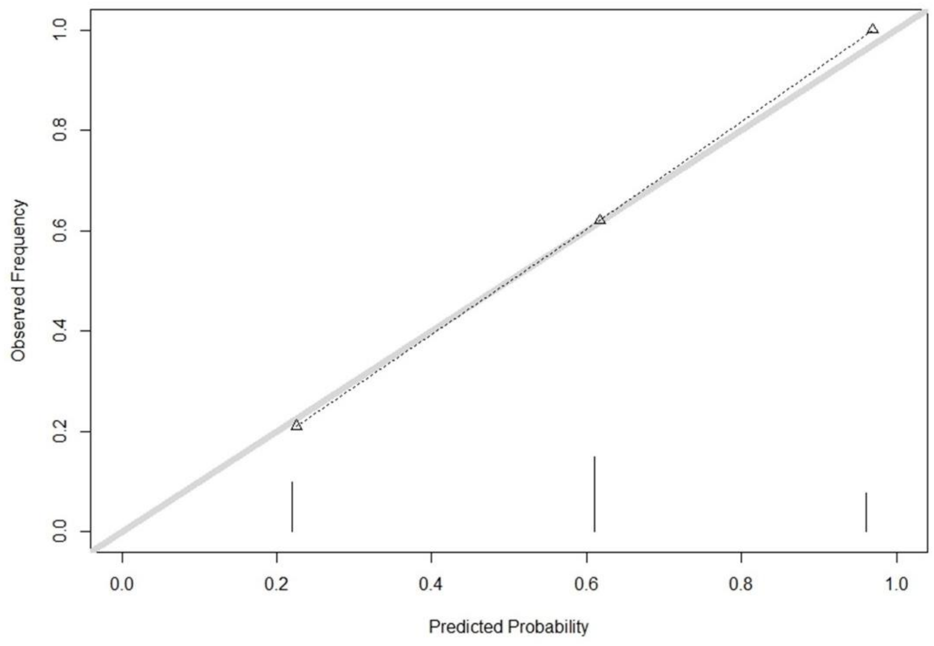

Performance measures of CT-based diagnostic prediction model without APOE genotype in the development dataset (n = 62) and following internal validation using the same dataset (n = 62; 2,000 bootstrap samples).

	Development	Internal validation
**Overall**		
Brier score	0·16	0·17
R^2 (Nagelkerke)	0·31	0·28
Akaike information criterion	65·03	68·90
**Discrimination**		
c statistic	0·82	0·82
Discrimination slope	0·32	0·31
**Calibration**		
Hosmer-Lemeshow test	χ^2 = 0·48, p = 0·92	χ^2 = 1·35, p = 0·72

Risk categories for lobar ICH associated with moderate/severe CAA using CT-based diagnostic criteria without APOE genotype

Predictors present		Predicted risk of moderate/severe CAA, %	Observed frequency of moderate/severe CAA (%) [95% CI]		Moderate/severe CAA probability
Subarachnoid haemorrhage	Finger-like projections				
-	-	23	4/19 (21)	[9-43]	Low
+	-	62	18/29 (62)	[44-77]	Medium
+	+	97	14/14 (100)	[78-100]	High

Cross tabulations of the index test results against the reference standard using the simplified Edinburgh (CT-only) CAA-associated lobar ICH diagnostic criteria

**Diagnostic criteria (index test)**	**Reference standard**		
**Subarachnoid haemorrhage**	**Moderate/severe CAA at post mortem**		
	Positive	Negative	Total
Positive	32	11	43
Negative	4	15	19
Total	36	26	62
**Diagnostic criteria (index test)**	**Reference standard**		
**Subarachnoid haemorrhage and finger-like projections**	**Moderate/severe CAA at post mortem**		
	Positive	Negative	Total
Positive	14	0	14
Negative	22	26	48
Total	36	26	62

Diagnostic test accuracy statistics for the simplified Edinburgh (CT-only) CAA-associated lobar ICH diagnostic criteria without APOE genotype

	Diagnostic criteria			
	Subarachnoid haemorrhage		Subarachnoid haemorrhage and finger-like projections	
**Sensitivity**	89	(73-96)	39	(24-56)
**Specificity**	58	(37-76)	100	(84-100)
**Positive likelihood ratio**	2·1	(1·3-3·3)	Inf	(NaN-∞)
**Negative likelihood ratio**	0·2	(0·1-0·5)	0·6	(0·5-0·8)
**Positive predictive value**	74	(59-86)	100	(73-100)
**Negative predictive value**	79	(54-93)	54	(39-68)
**Youden's Index**	0·47		0·39	

Data are percentage or ratio (95% confidence interval). NaN = not a number – calculation cannot be performed because one of the values includes a zero.

Unique MRC Brain Brank Network (BBN) numbers of included participants

BBN_7268	BBN_2561	BBN_14393	BBN_19602	BBN_24323
BBN_2519	BBN001.26127	BBN_19993	BBN_19601	BBN_24343
BBN_2514	BBN_2563	BBN_9507	BBN_19599	BBN_24528
BBN_15254	BBN_2570	BBN001.26492	BBN_19600	BBN_25059
BBN_2554	BBN_2573	BBN_7276	BBN_19598	BBN_24670
BBN_2524	BBN_15813	BBN_9504	BBN_19364	BBN_24531
BBN001.26092	BBN_4173	BBN_9502	BBN_19596	BBN_25060
BBN_2583	BBN_20599	BBN_9506	BBN_22228	BBN_25057
BBN_2526	BBN_2578	BBN_15215	BBN_20994	BBN_25058
BBN_3776	BBN_2582	BBN20615	BBN_20611	BBN001.26493
BBN_2535	BBN_20602	BBN_15815	BBN_20603	BBN001.26394
BBN_3769	BBN_3763	BBN_24305	BBN_22224	BBN001.26491
BBN_2527	BBN_3764	BBN_15224	BBN_24302	BBN001.26490
BBN_2539	BBN_3766	BBN_20992	BBN_22227	BBN001.26496
BBN_2543	BBN_3777	BBN001.26307	BBN_22223	BBN001.26499
BBN_2574	BBN_3775	BBN_15812	BBN_22226	BBN001.26719
BBN_2545	BBN_19994	BBN_15814	BBN_22626	BBN001.26722
BBN_2544	BBN_3781	BBN_15811	BBN_22627	BBN001.28404
BBN_2546	BBN_3782	BBN_19605	BBN_23394	BBN001.28405
BBN_19597	BBN_7627	BBN_19606	BBN_24303	BBN001.28408
BBN_2548	BBN_3787	BBN_19604	BBN_24304	BBN001.28411
BBN_2549	BBN_4167	BBN_19603	BBN_24306	BBN001.28416

Reference List

1. Newman GC. Clarification of abc/2 rule for ICH volume. *Stroke* 2007; **38**(3): 862.
2. van Swieten JC, Hijdra A, Koudstaal PJ, van Gijn J. Grading white matter lesions on CT and MRI: a simple scale. *J Neurol Neurosurg Psychiatry* 1990; **53**(12): 1080-3.
3. Sato S, Delcourt C, Heeley E, et al. Significance of Cerebral Small-Vessel Disease in Acute Intracerebral Hemorrhage. *Stroke* 2016; **47**(3): 701-7.
4. Charidimou A, Schmitt A, Wilson D, et al. The Cerebral Haemorrhage Anatomical RaTing inStrument (CHARTS): Development and assessment of reliability. *J Neurol Sci* 2017; **372**: 178-83.
5. Samarasekera N, Lerpiniere C, Fonville AF, et al. Consent for Brain Tissue Donation after Intracerebral Haemorrhage: A Community-Based Study. *PLoS One* 2015; **10**(8): e0135043.
6. Samarasekera N, Al-Shahi SR, Huitinga I, et al. Brain banking for neurological disorders. *Lancet Neurol* 2013; **12**(11): 1096-105.
